# Metal–Organic Frameworks (MOFs) for the Delivery of Small-Molecule Therapeutics

**DOI:** 10.1021/jacs.6c06915

**Published:** 2026-08-13

**Authors:** Pei-Hong Tong, Xi-Le Hu, Kai-Cheng Yan, Xiao-Shuai Yuan, Tony D. James, Hong-Yang Wang, Xiao-Peng He

**Affiliations:** † Key Laboratory for Advanced Materials and Joint International Research Laboratory of Precision Chemistry and Molecular Engineering, Feringa Nobel Prize Scientist Joint Research Center, School of Chemistry and Molecular Engineering, Frontiers Center for Materiobiology and Dynamic Chemistry, 47860 East China University of Science and Technology, 130 Meilong Rd, Shanghai 200237, China; ‡ National Center for Liver Cancer, the International Cooperation Laboratory on Signal Transduction, Eastern Hepatobiliary Surgery Hospital, Shanghai 200438, China; § Department of Radiation Oncology, Shanghai Pulmonary Hospital, 12476 Tongji University, No. 507 Zhengmin Road, Shanghai 200433, China; ∥ Department of Chemistry, 1555 University of Bath, Bath BA2 7AY, U.K.; ⊥ School of Chemistry and Chemical Engineering, Henan Normal University, Xinxiang 453007, China

## Abstract

As cutting-edge porous materials, the properties of metal–organic frameworks (MOFs) are governed by their metal nodes and organic linkers, as well as the assembly mode of these subunits. This allows the loading capacity of MOFs for small-molecule drugs to be readily tailored by rationally adjusting their structures and physicochemical properties. Furthermore, the introduction of additional functional groups into MOFs can enable the stimuli-responsive release of small-molecule drugs, thus rendering MOFs promising candidates for small-molecule drug delivery. With this perspective, we summarize the advances in the construction of MOF-based small-molecule drug delivery carriers and analyze the advantages and limitations of the various loading strategies for small-molecule drugs. We also introduce drug release mechanisms and their application for the targeted treatment of various human diseases. In particular, this perspective describes how the corresponding drug-loading strategies should be selected based on the inherent physicochemical properties of the small-molecule drugs and the specific biomedical application, in order to facilitate clinical translation.

## Graphical abstract

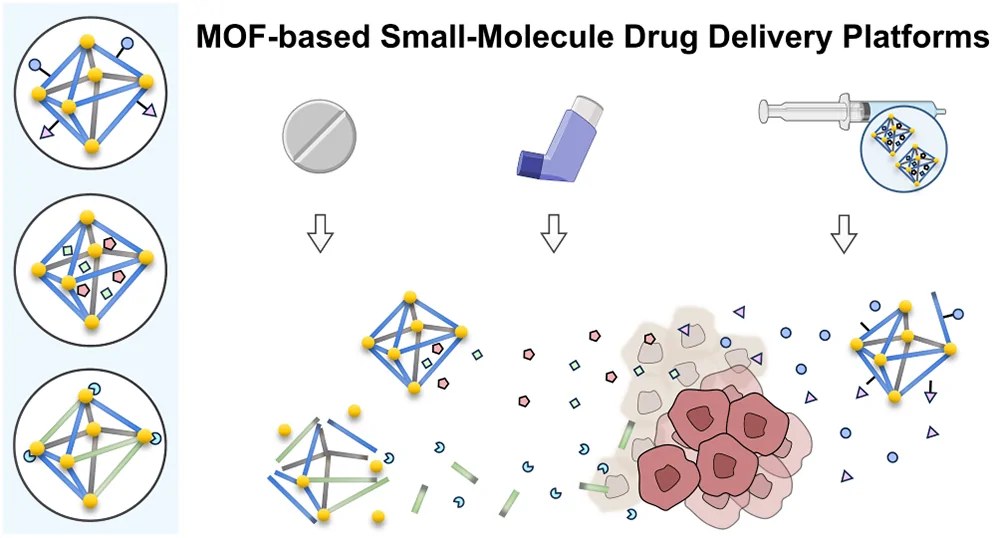

## 1 Introduction

The treatment of fatal human diseases such as cancer, metabolic diseases, and neurodegenerative disorders remains a significant challenge. Small-molecule drugs (molecular weight <1000 Da), as the main therapeutic agent, are widely used in clinical settings to treat these diseases.^1–3^ They have the advantages of broad applicability and well-established therapeutic mechanisms.^4^ Small-molecule drugs are often signal transduction inhibitors. For example, they can specifically block key signal transduction pathways required for tumor growth and proliferation to achieve therapeutic effects. Nevertheless, the inherent limitations of small-molecule drugs give rise to side effects including insufficient drug delivery to the target sites, issues related to drug degradation or solubility, poor cell membrane permeability, and off-target effects.^5,6^

Nanomedicine has seen rapid development in the past few decades to selectively deliver drugs to target tissues. Various nanomaterials have been used as drug delivery systems, such as metal nanoparticles,^7^ mesoporous silica,^8^ dendrimers,^9^ liposomes,^10^ and organic micelles.^11–13^ However, the aforementioned nanocarriers have limitations in biological applications. For example, organic materials suffer from low loading capacity and unsatisfactory control of drug release,^14,15^ and metal oxides suffer from adverse toxicity and nondegradability.^16,17^ Since MOFs were first explored as drug delivery carriers in 2006,^18^ small-molecule drug delivery systems based on MOFs have developed rapidly in recent years. As a kind of organic–inorganic hybrid material formed by self-assembly of metal ions/clusters and organic ligands,^19,20^ MOFs feature many advantages for drug delivery systems as follows: (1) MOFs generally have high porosity and large specific surface area, which are beneficial for improving the loading efficiency of small-molecule drugs.^21–23^ (2) MOFs have the characteristics of simple surface functionalization, controllable size, and morphology.^24^ (3) Small-molecule drugs are loaded into MOFs through various interactive forces such as hydrogen bonds, van der Waals forces, electrostatic forces, coordination bonds, π-π stacking, and covalent bonds. (4) MOFs can be stimuli-responsive drug delivery systems capable of releasing small-molecule drugs controlled by exogenous stimuli (*e.g*., temperature, pressure, and light) and endogenous stimuli (*e.g*., pH, ATP, glucose, ions, glutathione, and enzymes).^25–27^ MOFs are thus considered promising candidates for the delivery of small-molecule drugs. Although there have been reviews summarizing the application of MOFs in the field of drug delivery,^28–34^ they have failed to provide sufficient evaluation of the strategies for loading small-molecule drugs into MOFs, guidance on the selection of appropriate drug loading strategies based on the biomedical application scenarios and inherent properties of the small-molecule drugs. Moreover, the rapid emergence of novel design strategies and synthetic methodologies in this field further highlights the need for a comparative and analytical summary. Examples include the rapid preparation of MOFs via mechanochemical synthesis with little to no organic solvent consumption (i.e., a green, cost-effective method),^35,36^ the construction of full-component pharmaceutical MOFs using small-molecule drugs as building units,^37^ the screening of MOF carriers by artificial intelligence (AI) such as machine learning (ML),^38^ and the improvement of selective binding toward drugs via the rational design of biomimetic multivariate MOFs.^39,40^

This perspective aims to provide researchers with a systematic understanding of MOF-based small-molecule drug delivery carriers, aiding in the selection of appropriate drug loading strategies to capitalize on the structural advantages of MOF carriers in terms of drug loading capacity, thus minimizing premature drug leakage and inactivation. This will optimize the performance of MOF delivery systems and accelerate clinical applications. We will stress on the strategies for loading small-molecule drugs into MOFs, discuss the advantages and disadvantages of these strategies, their related drug release mechanisms, and their biomedical applications (Figure 1), thereby shedding light on the rationale by which to select MOF carriers and their loading strategies based on the properties of small-molecule drugs (*e.g.,* molecular size, stability, and functional groups). We will also discuss the remaining challenges in the clinical translation of MOF carriers and outline future development prospects in terms of MOF material biocompatibility, stability, targeting, repeatability, and the emerging role of AI. Cumulatively, these advances provide guidance for the rational design and construction of MOF-based drug delivery carriers.

**Figure 1:**
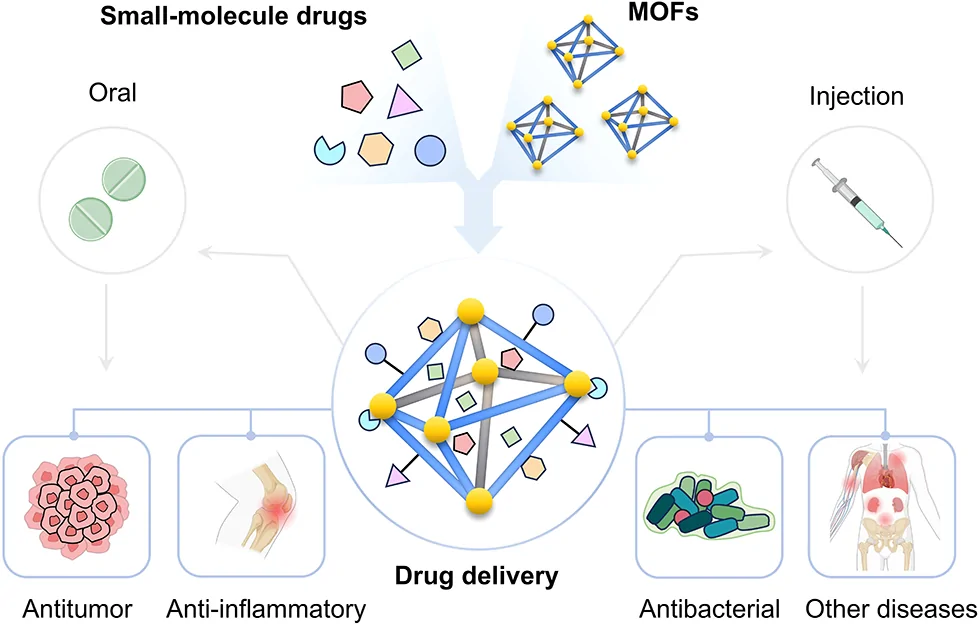
Schematic diagram of MOF-based small-molecule drug delivery platforms. MOFs act as nanocarriers to encapsulate a diverse range of small-molecule therapeutics for drug delivery. The MOF-based delivery platforms have been used for antitumor, anti-inflammatory, and antibacterial therapies, among other diseases.

## 2 Strategies for Loading Small-Molecule Drugs into MOFs and Their Applications in Disease Therapy

The concept of MOFs can be traced back to the work of Hoskins and Robson in 1989.^41^ Since then, a diverse class of MOFs have emerged, which are named according to the research institutions where they were first synthesized or based on their structural characteristics. Representative examples include Zeolitic Imidazolate Frameworks (ZIFs),^42,43^ characterized by zeolite-like topologies and imidazolate linkers; Materials of Institute Lavoisier (MILs) as developed by Férey and colleagues featuring diverse metal clusters and carboxylate linkers;^44^ University of Oslo (UiO-n), known for their exceptional stability arising from Zr_6_O_4_(OH)_4_ clusters;^45^ and Porous Coordination Networks (PCNs), developed at Texas A&M University with high porosity and functional tunability.^46,47^ MOFs are attractive as drug delivery carriers because of their excellent drug loading capacity and the tunability for controlled release of payloads.

Small-molecule drugs can be loaded into MOFs through a variety of strategies, which differ not only in loading mechanisms but also in experimental conditions.^48^ In general, these strategies can be classified into two-step and one-step methods. The two-step method is commonly conducted in liquids by exposing preformed MOFs to drug solutions, enabling surface adsorption, pore encapsulation, or covalent chemistry. In addition, pore encapsulation can also be achieved using mechanochemical methods, such as grinding or liquid-assisted grinding, to reduce organic solvent consumption and accelerate the loading process. Alternatively, one-step methods include *in situ* encapsulation during MOF crystallization and the use of drugs as building units in MOF structures through coordination chemistry. These two strategies can be implemented via room-temperature rapid synthesis, mechanochemical methods, or solvothermal methods.

The choice of a drug loading strategy determines the interactive manner between drugs and MOF carriers, and the strength of such interactions is influenced by parameters of MOFs such as pore structure, surface chemistry, and metal coordination environment.^49^ Failure to select a suitable loading strategy matched to the molecular size, hydrophobicity, functional groups, and chemical stability of small-molecule drugs can lead to issues such as low drug loading capacity, drug leakage, carrier structural collapse, and premature drug inactivation in physiological environments. Meanwhile, the adaptability of loading strategies requires trade-offs tailored to specific biomedical scenarios. For diverse applications including blood circulation, tumor-targeted drug delivery, and local antibacterial therapy, delivery systems impose vastly different requirements on structural stability, sustained or stimuli-responsive drug release profiles, drug loading capacity, and biocompatibility, leading to substantial discrepancies in identifying an optimal loading protocol.^50^ As a consequence, screening loading strategies based on the intrinsic physicochemical properties of drugs according to clinical demands is crucial for the rational design of MOF-based drug delivery platforms.

### 2.1 Drug-Loaded MOFs Constructed by Surface Adsorption

Surface adsorption is the simplest strategy for loading small-molecule drugs (Figure 2a).^51^ This strategy relies on noncovalent interactions between drugs and the surface sites of MOFs, including (1) van der Waals forces, which arise from the interaction between transient or permanent dipoles induced by quantum effects with a relatively low binding strength. (2) Hydrogen bonding interactions, which are supramolecular forces formed between electron-deficient hydrogen atoms and electronegative atoms (or electron-rich aromatic ring structures). The strength of the interaction depends on the acidity or basicity of the donor and acceptor, and the interaction is stronger than that of van der Waals forces. (3) π–π stacking interactions, which arise from the electrostatic correlation between electron-deficient edges and electron-rich centers between aromatic rings. The strongest interaction occurs in parallel geometries, and the strength is between that of van der Waals forces and hydrogen bonds. (4) Electrostatic interactions, which arise from the Coulomb attraction between groups with opposite charges; this form of binding is the strongest.^52^ The preparation process for this strategy involves adding activated MOFs to a drug solution, followed by drug loading through constant-temperature stirring, oscillation, or static immersion. This is beneficial for drug molecules containing labile groups (such as ester bonds and peptide bonds). It preserves the original molecular structure and pharmacological activity of the drugs and also simplifies the process of formulation development.

**Figure 2:**
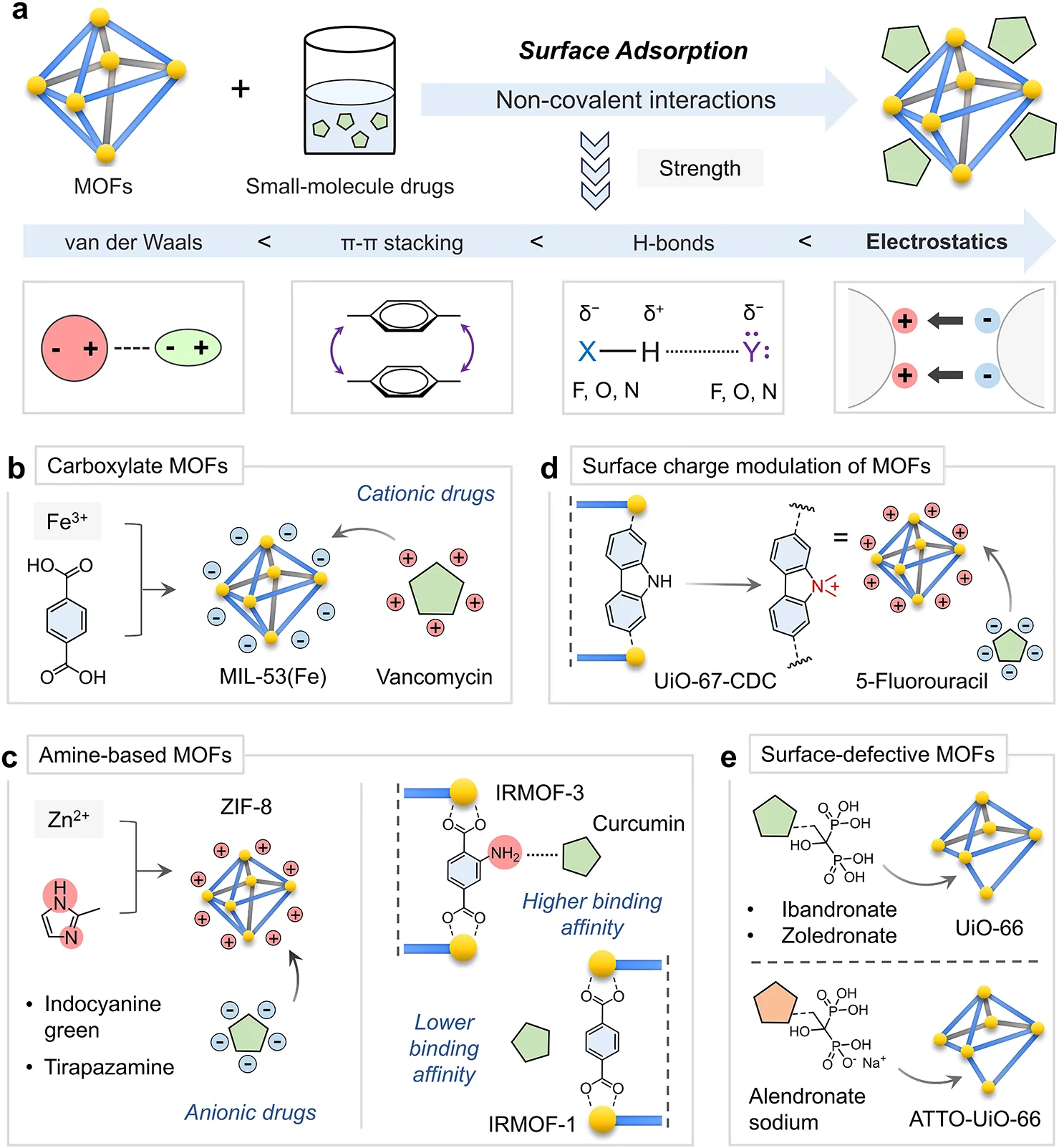
Schematic illustration of drug-loaded MOFs constructed by surface adsorption. a) Noncovalent interaction-mediated surface adsorption for drug loading onto MOFs. b) Van/MIL-53­(Fe) nanocomposite, prepared by loading Van onto the MIL-53­(Fe) carrier for sustained antibacterial applications with low cytotoxicity.^54^ c) Loading ICG and TPZ onto the ZIF-8 carrier for the synergistic photodynamic therapy (PDT) and chemotherapy of tumors; amino-functionalized MOF (IRMOF-3) as a carrier for the antitumor drug curcumin.^57^ d) Design of *N*-methylating UiO-67-CDC to obtain cationic UiO-67-CDC-(CH_3)2_ for the efficient loading of 5-Fu.^59^ e) Surface-defective Zr-based MOFs with exposed metal sites that enhance the loading of drugs containing phosphate or phosphonate groups.^62,63^

The drug loading capacity of MOFs is mainly affected by the type and quantity of functional groups on their surface. Functional groups such as −NH_2_, −COOH, −OH, and pyridyl groups can effectively regulate the adsorption affinity of MOFs for drugs. Generally, carboxylate MOFs are more conducive to the adsorption of cationic drugs.^53^ For example, MIL-53­(Fe) was used to load Vancomycin (Van).^54^ The negative charges originating from −COOH groups of the organic ligands on the MOF surface interacted electrostatically with the positively charged Van (Figure 2b) to drive the adsorption of drug molecules; an effective drug-loading efficiency of 20 wt % was achieved. In a bacterium-infecting microenvironment, MIL-53­(Fe) slowly degraded to release Van, achieving an antibacterial efficiency of up to 99% against methicillin-resistant*Staphylococcus aureus* (MRSA). It is worth noting that the molecular weight of Van (1449 Da) exceeds the definition of a small-molecule drug defined in this study, and is used only for mechanistic explanation.

Amino-based MOFs are better suited for anionic drugs and exhibit enhanced binding affinity.^55,56^ For example, ZIF-8 exhibited a positive zeta potential. Upon coloading with indocyanine green (ICG) and tirapazamine (TPZ) (Figure 2c), the zeta potential decreased to −12 mV, with loading efficiencies of 9 and 17 wt %, respectively.^57^ In the acidic tumor microenvironment, the imidazole ligands were protonated to cleave the Zn–N coordination bond, which in turn degraded ZIF-8 for drug release. However, the therapeutic effect of this system relies on a multistep cascade activation process, making it difficult to achieve stable and reproducible therapeutic outcomes. In another example, the zinc-based amino-functionalized MOF (IRMOF-3) was used as a carrier for the antitumor drug curcumin (Cur) (Figure 2c).^58^ Compared with the isomorphic amine-free counterpart (IRMOF-1), IRMOF-3 had a higher drug-loading capacity (55% vs 49%) and a slower Cur release rate. The superior performance of IRMOF-3 was attributed to the affinity between its side-chain amino groups and Cur, which enhanced the drug loading capacity and decreased the drug release rate.

The surface charge of MOFs can also be adjusted by tuning the electronic structure of the ligands. For example, 9H-carbazole-2,7-dicarboxylic acid (9H-2,7-CDC) was used a ligand to synthesize carbamoyl-functionalized UiO-67-CDC. UiO-67-CDC was efficiently cationized via *N*-methylation. The cationic framework of UiO-67-CDC-(CH_3_)_2_ exhibited strong electrostatic interactions with electron-rich 5-FU, resulting in a high drug-loading efficiency of 57 wt % (Figure 2d).^59^ 5-Fu/UiO-67-CDC-(CH_3_)_2_ could effectively and persistently release drugs in a simulated blood environment (pH 7.4). Another noteworthy feature was that UiO-67-CDC displayed a unique fluorescence turn-on response toward 5-FU. These results guide the design of surface-engineered MOF for drug delivery and fluorescence-based sensing applications. In addition, postsynthetic modification with agents such as alginate (negatively charged) and chitosan (positively charged) can introduce the corresponding charges to MOFs.^60,61^ These modifications alter the surface chemistry of MOFs, enabling electrostatic interactions with oppositely charged drugs to ultimately achieve efficient drug loading.

Surface-defective Zr-based MOFs can significantly enhance the loading capacity of drugs containing phosphate or phosphonate groups (Figure 2e). A systematic comparison of the loading behavior of 12 bioactive molecules in UiO-66 indicated that those containing phosphate/phosphonate groups, such as ibandronate (Iband) and zoledronate (ZOL), achieved a high loading efficiency of 80%–100% in defective UiO-66. In a control experiment, the same drugs were hardly loaded into nondefective UiO-66. Compared to nondefective UiO-66 with intact crystal lattices, defective UiO-66 possesses larger specific surface area and more unsaturated metal sites, while the exposed sites significantly enhance the drug loading capacity. Molecules without these charged groups (*e.g.,* adenosine and cytidine) exhibited an extremely low loading efficiency of ∼1 wt %.^62^ Mechanistic studies indicated that the Zr sites exposed by defects can form strong Coulombic interactions with the phosphate/phosphonate groups of drugs. In a second example, Huang and coworkers synthesized a colloidally stable fluorescent MOF (ATTO-UiO-66) via a defect engineering strategy for high-efficiency loading of alendronate sodium (AL), with a loading capacity of ∼30 wt %. Under an acidic condition of pH 5.0, the ATTO-UiO-66 carrier achieved sustained drug release for up to 90 h.^63^

Overall, this strategy is more suitable for local drug delivery or short-range delivery scenarios, such as wound infection or local pain management. Although the electrostatic adsorption strategy offers high drug loading efficiency and mild preparation conditions, the resulting drug–MOF interactions are inherently weak. Upon entering the physiological environment, drugs tend to leak prematurely due to the interference of high concentrations of ions and biomacromolecules. Another limitation is that this loading strategy is not suitable for labile drugs, as drug molecules tend to be degraded before reaching the target tissue. This strategy is considered more suitable for loading water-soluble ionic drugs for the treatment of diseases such as wound infections. When further combined with hydrogels, medical dressings, or functional coatings, MOFs can enable sustained local drug release. Consequently, drugs with anti-inflammatory, analgesic, or antibacterial functions are generally better suited to this strategy.

### 2.2 Drug-Loaded MOFs Constructed by Covalent Chemistry

In general, the surface of MOFs can be tailored with various functional groups (*e.g*., −COOH, −NH_2_, and −OH)^64^ that can be exploited to form covalent bonds with reactive groups of active drugs. Compared with the surface adsorption strategy, covalent attachment effectively minimizes unwanted loss of drug molecules *in vivo*, and conjugation strategies that incorporate stimuli-responsive linkers can undergo cleavage upon exposure to specific chemical triggers. Figure 3a summarizes the chemical bonds commonly used as covalent linkages in MOF–drug systems.^65–69^ When selecting an appropriate covalent linkage, one should consider the retention of conjugation stability during the *in vivo* circulation process prior to drug release at the target site.

**Figure 3:**
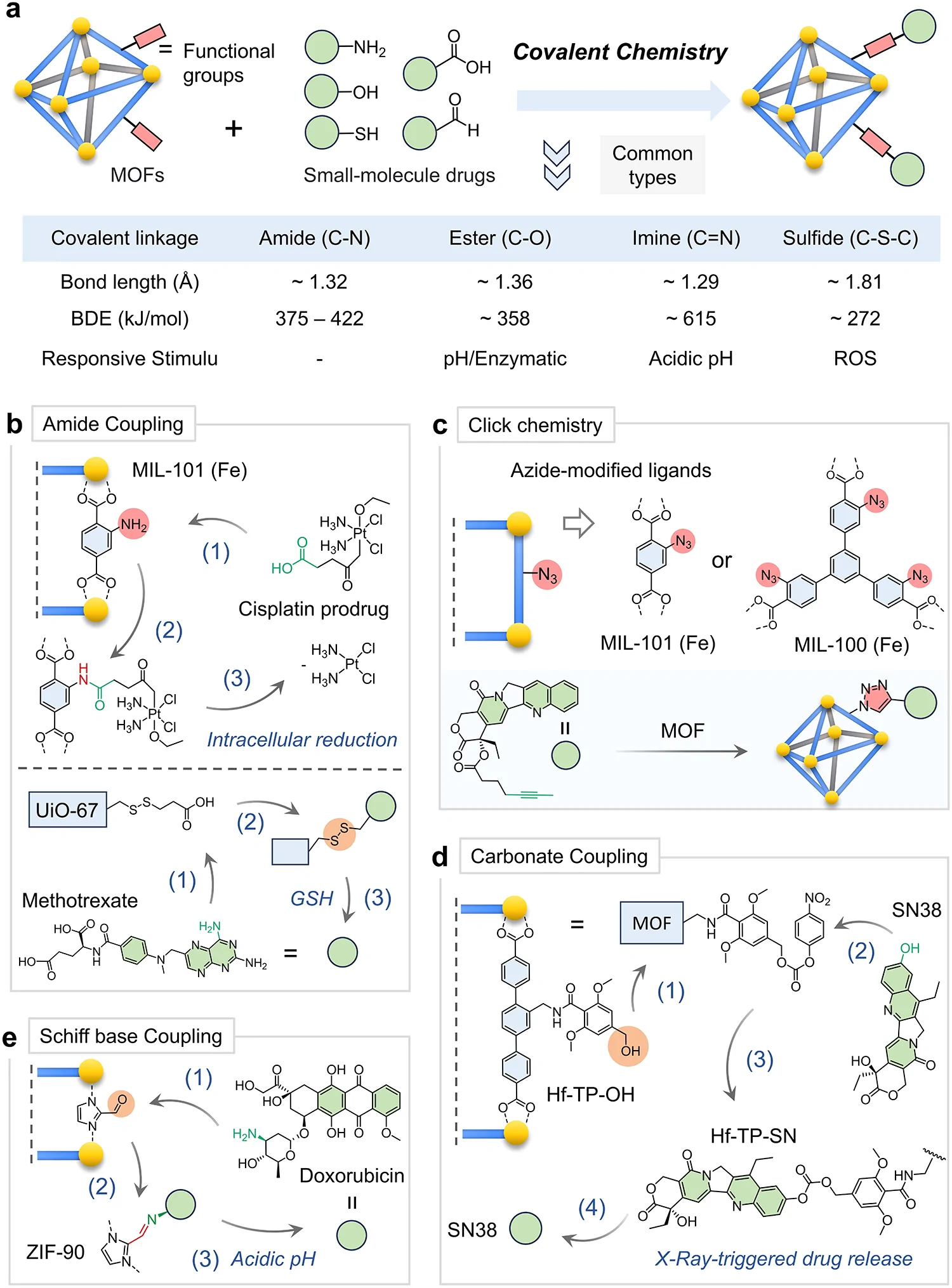
Schematic illustration of drug-loaded MOFs constructed by covalent chemistry. a) Drug loading onto MOFs via reactions between functional groups on MOF surfaces and complementary groups on drugs, and representative covalent linkages in MOF–drug conjugates. b) ESCP/MIL-100­(Fe) composite, prepared by loading ESCP onto the MIL-100­(Fe)-NH_2_ carrier, for antitumor therapy; methotrexate–conjugated UiO-67 for intracellular GSH–triggered drug release.^71,74^ c) Coupling of CPT to azide-functionalized MIL-101­(Fe) or MIL-100­(Fe) via click chemistry.^74^ d) Construction of Hf-TP-SN nanoparticles for X-ray responsive SN38 drug release.^79^ e) ZIF-90 with abundant aldehyde groups as a carrier for the antitumor drug DOX.^81^

This strategy is suited for small-molecule drugs that have the potential for prodrug derivatization without compromising pharmacodynamic properties.^70^ Mature strategies for prodrug design can be simply exploited to construct covalent drug delivery systems based on MOFs. For example, amino groups were incorporated into the framework by doping terephthalic acid with 2-aminoterephthalic acid during the assembly of MIL-101 (Fe). Then a cisplatin prodrug (ethoxysuccinato–cisplatin, ESCP) was conjugated to the ligands via amide bonds (Figure 3b).^71^ The conversion rate of modifiable amino sites in the framework reached 40.2%, with a drug-loading efficiency of 12.8 wt %. MIL-101­(Fe) degraded under physiological conditions, releasing ESCP, and the drug release kinetics were modulated by coating MIL-101­(Fe) with a SiO_2_ shell, extending the release half-life from 1.2 to 14 h. The applicability of this delivery system for antitumor therapy was verified *in vitro* using HT-29 human colon adenocarcinoma cells. Moreover, the SiO_2_ coating could be replaced with organic agents such as alginate, polyvinylpyrrolidone, and chitosan to further improve biocompatibility.^72^

Click chemistry is also a simple and efficient strategy for covalent modification of MOFs.^73^ Botella and coworkers used MIL-101­(Fe) and MIL-100­(Fe) as carriers, and converted the MOF surface amino groups to azide groups. The azido MOFs were then reacted with alkyne-modified camptothecin (CPT) via a Cu­(I)-catalyzed azide–alkyne cycloaddition reaction (CuAAC) to achieve efficient covalent fixation of CPT (Figure 3c). The CPT loading capacity of MIL-101­(Fe) and MIL-100­(Fe) was 18.0 and 9.2 wt %, respectively. This difference was attributed to a larger specific surface area of MIL-101­(Fe) (1824 m^2^/g) than that of MIL-100­(Fe) (880 m^2^/g).^74^ Meanwhile, due to the positive charge of CPT, the CPT-loaded MOFs exhibit enhanced cellular internalization. The MOFs are pH sensitive, with a 4-fold increase in drug release at pH 5 with respect to that at neutral pH, facilitating intracellular drug release in the acidic endosomes and lysosomes. Although the MOF frameworks degraded under acidic conditions, CPT retained covalently bound to the organic ligands—a property that could compromise the therapeutic efficacy of the drug molecules.

Despite these advances, the irreversible covalent coupling of drug molecules with organic ligands requires operable triggering conditions to release the drugs. To achieve controlled-release of drugs, it is important to introduce environmentally sensitive chemical bonds, such as reduction-sensitive disulfide bonds,^75^ light-sensitive O-nitrobenzyl linkages,^76^ and acid-sensitive hydrazone and imine bonds, to MOFs.^77^ For example, Rohani and coworkers constructed a functionalized MOF (MOF–S–S–COOH) containing a disulfide-bonded carboxyl side chain on UiO-67 via a three-step postsynthetic modification, which was subsequently covalently coupled with methotrexate (MTX) (Figure 3b). The release of MTX from the MOF in PBS (pH 7.4) reached 74% in 28 h in the presence of 10 mM GSH; this number decreased to 28% in the absence of GSH.^78^ In another example, Lin and coworkers developed a novel MOF-based prodrug (Hf-TP-SN), where its organic ligands form covalent linkages with 7-ethyl-10-hydroxycamptothecin (SN38).^79^ Under X-ray irradiation, the electron-dense Hf12 SBU in Hf-TP-SN acted as a radiosensitizer, enhancing the production of hydroxyl radicals (Figure 3d). These hydroxyl radicals induced hydroxylation and 1,4-elimination of 3,5-dimethoxybenzyl carbonate, thereby facilitating the release of SN38 from Hf-TP-SN. The release efficiency was 5-fold higher than that of traditional small-molecule prodrugs, allowing the effective inhibition of tumor growth in murine models of both colon and breast cancer.

For drugs containing amino-reactive sites, covalent conjugation using dynamic Schiff base bonds formed under mild conditions is an ideal option.^80^ As a member of the ZIF family, ZIF-90 was chosen as the model framework due to its simple synthetic route and the easy functionalization of the imidazole-2-carboxaldehyde (ICA) ligand within its framework. Doxorubicin (DOX) was covalently coupled to the surface of the ZIF-90 carrier by the Schiff base reaction between the amino group in DOX and the aldehyde group of ICA ligands (Figure 3e), achieving a drug loading capacity of 8 wt %.^81^ Under a physiological environment (pH 7.4), less than 17% of DOX was released within 24 h, suggesting resistance to premature leakage *in vivo*. In contrast, ∼70% was released within 12 h under a weakly acidic pH of 6.5, and over 90% was released within 8 h under a stronger acidic condition (pH 5.0), suggesting the potential of the MOF for site-specific drug release in the acidic tumor site. DOX/ZIF-90 exhibited minimal cytotoxicity toward normal 3T3 cells, maintaining an 87% cell viability even at 100 μg mL^–1^. In contrast, the MOF markedly supressed the proliferation of 4T1 breast cancer cells in a dose-dependent manner, with cell viability decreasing from 80% to 30% as the concentration increased from 25 to 100 μg mL^–1^.

In addition to the aforementioned loading strategies, therapeutic drugs can also be covalently conjugated with a bridging ligand, and then coordinated with metal ions to construct MOF vehicles. For example, Methotrexate (MTX) was covalently conjugated to tannic acid (TA) in a 2:1 molar ratio via esterification. Then, the conjugate was attached to MOFs through the strong coordination between TA and Fe^3+^. The MOF surface was then modified with hyaluronic acid (HA) to enhance its targeting capability. The resulting MTX/MOFs exhibited an extremely high drug loading capacity (45 wt %) and sustained drug release (∼50% release within 24 h).^82^ *In vivo* experiments confirmed that the as-prepared MOFs enhanced the antirheumatic efficacy of MTX.

Although the covalent-chemistry strategy offers more stable drug immobilization than other methods, it still has several limitations for practical applications: (1) many drugs cannot be modified using this strategy due to a lack of modifiable functional groups; (2) covalent conjugation requires relatively complex procedures such as multiple synthetic and purification steps; (3) most MOFs are prone to coordination bond breakage when undergoing postsynthetic chemical reactions. Therefore, selecting MOF carriers with good framework stability (*e.g.,* Zr-based MOFs) are necessary.^83,84^ Therefore, future research should focus on stimuli-responsive release, that is, drugs can be covalently bonded to MOFs via environmentally sensitive chemical bonds. Upon reaching target sites, the corresponding chemical bonds can be cleaved, thereby achieving on-demand release of drugs to improve therapeutic efficacy. Despite the numerous design strategies available for stimulus-responsive MOFs, none have succeeded clinically to date. A major reason for this is that the intensity, spatiotemporal distribution, and individual differences of stimulative signals in the human body are far more complex than the simplified scenarios typically tested *in vitro* or in animal models.^85^

### 2.3 Drug-Loaded MOFs Constructed by Pore Encapsulation

Since MOFs possess pores tunable from microporous to mesoporous, many types of drugs can be accommodated inside the pores. Pore encapsulation denotes the encapsulation of therapeutic agents into the framework of presynthesized MOFs via liquid impregnation or solid–solid mechanical methods.^86–88^ For this strategy to be successful, the molecular size of drugs should be smaller than the pore size of MOFs. Typically, drugs are encapsulated into the framework through noncovalent interactions and other host–guest interactions (Figure 4a). In addition, the pore surfaces of MOFs can be modified to adjust hydrophobicity/hydrophilicity and functional groups, thereby enhancing interactions with drug molecules. Since the porous structure of MOFs provides a physical barrier around the drugs, it can effectively prevent drug degradation. Therefore, compared with surface adsorption and covalent chemistry strategies, this approach is more suitable for *in vivo* drug delivery.

**Figure 4:**
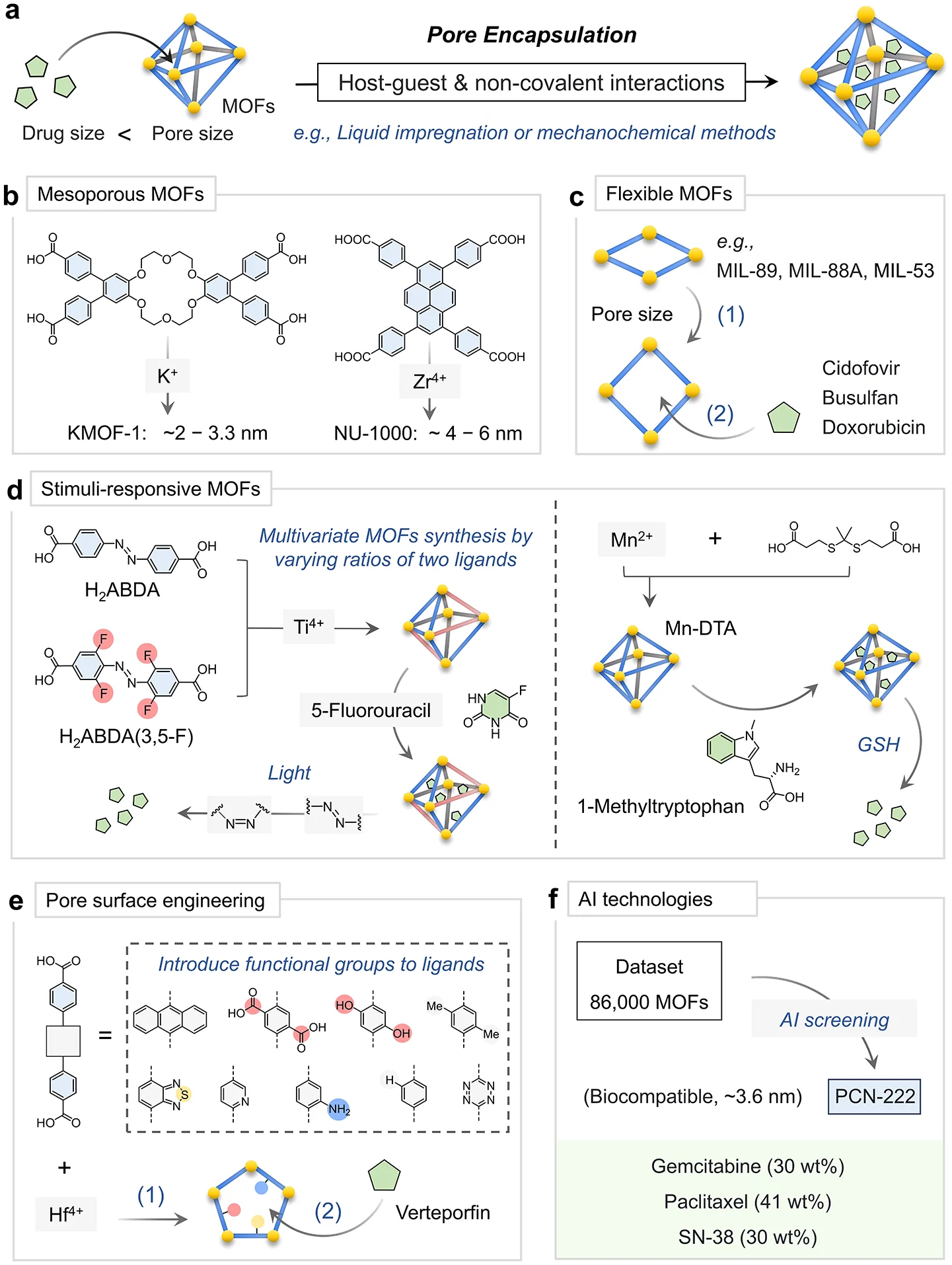
Schematic illustration of drug-loaded MOFs constructed by pore encapsulation. a) Schematic illustration of pore encapsulation, a process that incorporates drug molecules smaller than the MOF pore size into the presynthesized MOFs. b) Structure of representative mesoporous MOFs, KMOF-1 and NU-1000, capable of encapsulating relatively large-size drugs.^89,90^ c) Flexible MOFs allowing for reversible pore expansion/contraction to dynamically accommodate drugs with diverse sizes and shapes.^95^ d) Design strategy for fabrication of multivariate MOFs using varying ratios of photoactivated ligands H_2_ABDA and H_2_ABDA­(3,5-F); multifunctional 1-MT/Mn-DTA nanosystem for starvation, oxidation improved indoleamine 2,3-dioxygenase-blockade immunotherapy.^98,99^ e) Use of pore surface engineering via introducing functional groups to ligands to tailor the cavity microenvironment of biomimetic multivariate MOFs for the loading of verteporfin.^101^ f) Identification of MOFs suitable for encapsulation of gemcitabine, paclitaxel and SN-38 using artificial intelligence/machine learning.^102^

For drugs with relatively large molecular sizes or rigid planar structures (*e.g.,* DOX), mesoporous MOFs (such as NU-1000,^89^ KMOF-1,^90^ and MIL-101 ^91^) are suitable carriers (Figure 4b). While rigid pores cannot accommodate drugs with mismatched shapes, the use of flexible MOFs (*e.g.,* MIL-53 ^92^ and MIL-88 ^93,94^ ) can enable reversible structural transformations during drug adsorption, a phenomenon known as the breathing effect, where the cell volume increases by 50% to 230% without bond breakage. This adaptive behavior allows the pores to expand upon drug entry and contract after drug release, enabling dynamic encapsulation of drugs of varying sizes. These flexible MOFs were used to load small-molecule drugs (Figure 4c), including antitumor drugs (busulfan and DOX) and antiviral drugs (3′-azido-3′-deoxythymidine triphosphate (AZT-TP) and cidofovir). Specifically, MIL-89 was used for accommodating busulfan (9.8 wt %) and cidofovir (14 wt %), and MIL-88A for AZT-TP (0.6 wt %) and busulfan (8.0 wt %).^95^ These MOFs have also proved to have good biodegradability.

MOFs with pore sizes smaller than 2 nm are preferential for delivering drugs including ibuprofen, aspirin, and 5-FU. For example, using the hydrophilic aspirin and hydrophobic ibuprofen as model drugs, the loading and release behavior of the following three biocompatible MOFs were evaluated: hydrophobic microporous UiO-66 (pore size 8–11 Å, window 5–7 Å), moderately hydrophilic microporous MIL-127 (hydrophobic channel ∼6 Å, hydrophilic cage ∼10 Å, window ∼3 Å), and hydrophilic mesoporous MIL-100 (mesoporous cage 25–29 Å, window 4.8–8.6 Å).^96^ Loading efficiency was found to mainly depend on the geometric matching between the pore size of MOFs and the drug, as well as a hydrophilicity–hydrophobicity balance. UiO-66 exhibited the highest ibuprofen loading efficiency (35.5 wt %) due to its suitable pore size and strong hydrophobicity. Although MIL-100 had the largest pore volume, only part of its large pores was occupied by drugs, resulting in a moderate loading. MIL-127 showed the lowest loading capacity due to its excessively small pore windows and narrow channels. The release dynamics was dominated by Higuchi diffusion, with the release rate in an order of MIL-100 (complete release in 1–3 days) > UiO-66 (1–7 days; hydrophilic drugs were released faster) > MIL-127 (4–6 days; ibuprofen exhibited zero-order constant release rate). In addition to the traditional liquid impregnation method, Navarro and coworkers loaded 5-FU onto Zn-based MOFs using a mechanochemical method (ball-milling).^97^ This method was solvent-free and much faster than the traditional immersion loading method. This ball-milling approach does not compromise the MOF structure, enabling rapid drug encapsulation.

Morris and coworkers constructed six visible-light-activated multivariate MOFs, whose drug loading capacity and release rate were systematically optimized by adjusting the linker ratio between 4,4′-azobenzene dicarboxylic acid (H_2_ABDA) and 4,4′-(diazene-1,2-diyl)­bis­(3,5-difluorobenzoic acid) (H_2_ABDA­(3,5-F)) (Figure 4d).^98^ Computational analysis suggested that the tetrahedral/octahedral pore windows formed between H_2_ABDA and H_2_ABDA­(3,5-F) at a molar ratio of 2:1 favor 5-FU encapsulation. The “multivariate” MOF formed between the two ligands was shown to significantly enhance the loading capability compared to those formed by either ligand alone (pure H_2_ABDA or H_2_ABDA­(3,5-F)). Under green light irradiation, photoisomerization of azobenzene initiated MOF disassembly, and the drug release rate increased with the proportion of fluorinated ligands. The MOF containing 100% H_2_ABDA­(3,5-F) achieved a maximum release rate of 0.9% of the total cargo per min (77% released in 2 h), offering an efficient design strategy for photocontrolled MOFs as delivery carriers. However, the *in vivo* application of light-responsive MOFs is still limited by poor tissue penetration of light, which makes activation at deep-seated lesions difficult. In another example, a mesoporous MOF (Mn-DTA) with an average pore size of 8.6 nm was constructed using the ROS-responsive ligand 5,5-dimethyl-4,6-dithionanoic acid (DTA) coordinated with Mn^2+^ to deliver 1-methyltryptophan (with a loading efficiency of 13.5 wt %) that blocks the immune checkpoint protein indoleamine 2,3-dioxygenase, thus activating immune response and reducing immune tolerance (Figure 4d).^99^ However, a key limitation lies in the significant heterogeneity in redox state of different tumor phenotypes, which makes the efficacy of drug release unpredictable.

Besides pore size matching, the chemical properties of the pore surface can also affect the binding affinity and stability of drugs. By introducing functional groups (−NH_2_, −OH, −COOH, *etc.*) to organic ligands, the hydrophobic/hydrophilic balance and hydrogen bond donor/acceptor distribution within the MOF pore can be modulated, thereby enhancing the selective binding of drugs.^100^ By enumerating all possible combinations of nine functional ligands, Li and coworkers incorporated biomimetic binding cavities and tunability into the reticular structures of MOFs (Figure 4e).^101^ Using a combinatorial approach, a total of 1089 MOFs—including both single-component and multicomponent examples—were systematically optimized to enhance the pore-encapsulation efficacy of verteporfin (VP, a clinically approved photodynamic therapy drug). *In vitro* studies on various cancer cell lines indicated that photodynamic therapy using bMTV-MOFs (A1F6H3 or A3D1H6) resulted in a 23- to 68-fold improvement in therapeutic efficacy compared with the pristine MOFs.

Despite significant progress, researchers rely on experimental expertise and chemical intuition to discover and design drug delivery carriers. Against this background, Fairen-Jimenez and coworkers used artificial intelligence (AI) to screen 86,000 MOFs and identify a biocompatible PCN-222 carrier (1.7 nm triangular micropores and 3.6 nm hexagonal mesopores).^102^ Advanced computational techniques were then employed to evaluate the encapsulation of three chemotherapeutic drugs, gemcitabine, paclitaxel and SN-38, within PCN-222, achieving drug loading rates of 30, 41, and 30 wt %, respectively (Figure 4f). This system remained stable in an aqueous solution at 4 °C for 12 months without obvious structural degradation or loss of therapeutic efficacy. In a mouse model of pancreatic cancer, the authors demonstrated that intravenous systemic administration of the MOFs effectively inhibited tumor growth by reducing the tumor size by 74% after 28 days of treatment. This study confirms the potency of using AI to promote the screening of functional MOF carriers for practical use.

The pore encapsulation strategy is drug-friendly, mild, and compatible with temperature-sensitive molecules. Moving forward, the modular characteristics of MOFs can be fully exploited to enable precise design across the following two dimensions: (1) precisely tuning the pore size (from micropores to mesopores) by selecting organic ligands of different lengths to accommodate drugs of varying molecular sizes; (2) introducing functional groups (*e.g.,* −NH_2_, −OH) onto organic ligands to optimize the chemical microenvironment within MOF cavities, enhance affinity for small-molecule drugs, and thereby improve drug loading capacity and stability.

### 2.4 Drug-Loaded MOFs Constructed by *In Situ* Encapsulation

The *in situ* encapsulation strategy capitalizes on the direct incorporation of drugs during the synthesis of MOFs (Figure 5a). Compared with the pore encapsulation strategy, the *in situ* encapsulation strategy has significant advantages in terms of drug distribution uniformity and loading efficiency, and can overcome the limitations of preformed MOF pore size on guest molecules. *In-situ* encapsulation requires that the temperature, pH, and solvent environment for the synthesis of MOFs do not affect the activity of drugs. Most high-temperature hydrothermal and solvothermal synthetic approaches lead to drug inactivation. Therefore, ambient and mild syntheses are required for labile drug molecules. Currently, MOFs that can be synthesized under mild conditions mainly include ZIF-8, ZIF-90, ZIF-67, and HKUST-1.^103^

**Figure 5:**
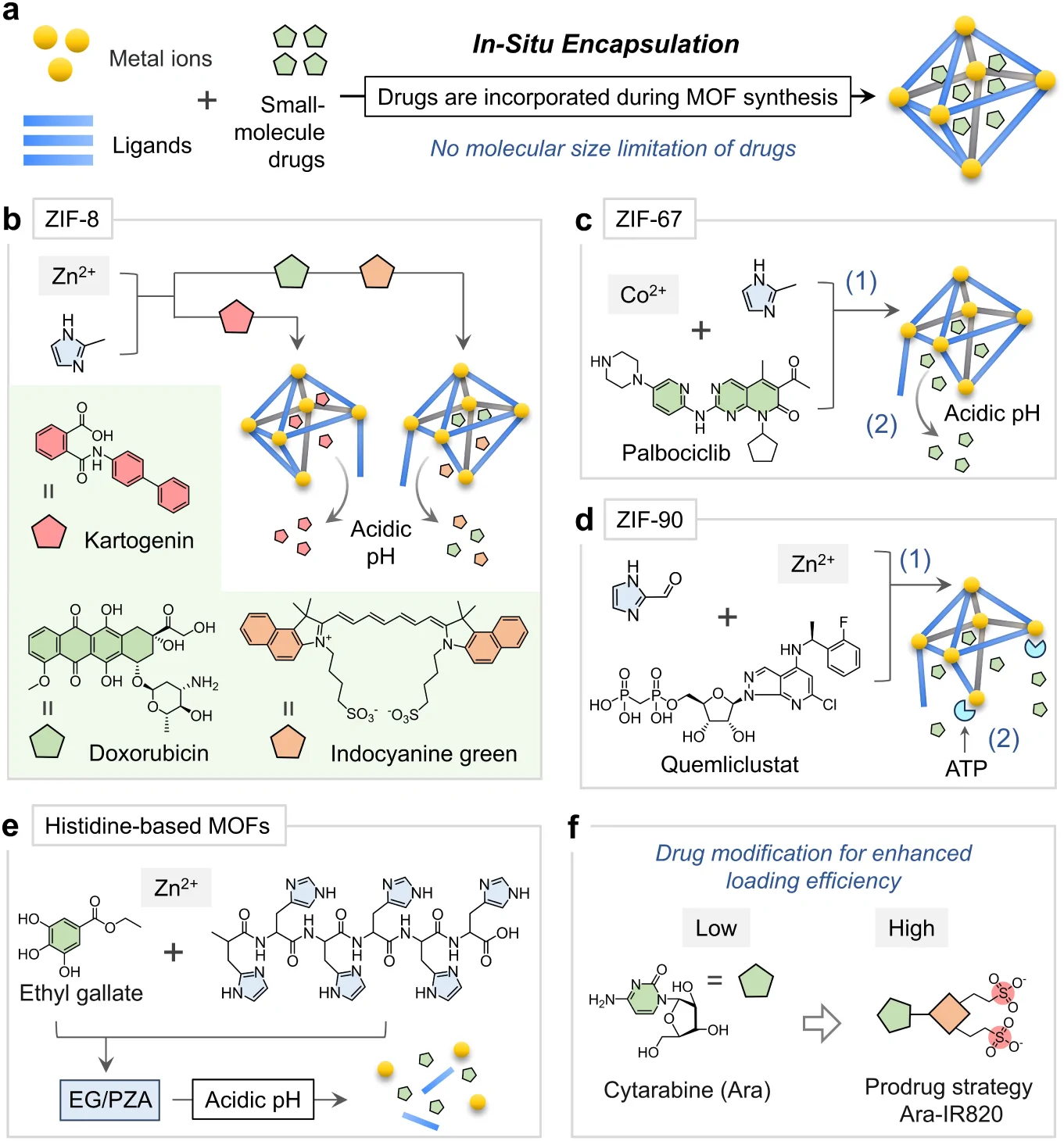
Schematic illustration of drug-loaded MOFs constructed by *in situ* encapsulation. a) *In situ* encapsulation strategy for incorporating small-molecule drugs during MOF crystallization. b) DOX/ICG/ZIF-8 nanocomposite, prepared by encapsulating DOX and ICG into the ZIF-8 carrier, for guided breast cancer therapy; self-reinforced KGN/ZIF-8-based nanogel alleviates osteoarthritis by sustained drug release.^105,106^ c) Senescence-primed ferroptosis enabled by a Pal/ZIF-67 platform for enhanced cancer therapy.^108^ d) ATP-responsive AB680/ZIF-90 with facile postmodification enhances therapeutic outcomes of triple-negative breast cancer.^109^ e) EG was loaded into a pH-responsive MOF assembled from Zn^2+^ and His6 ligands for osteoarthritis therapy.^110^ f) Converting poorly loadable Ara into an Ara-IR820 prodrug to achieve high drug loading by strengthening interactions with ZIF-8 for chemo-photothermal therapy.^114^

ZIF-8 can be synthesized at room temperature in neutral aqueous solution or methanol under extremely mild conditions, and is widely applied to loading of various drugs.^104^ Once loaded into the ZIF-8 framework, the cargo cannot diffuse freely due to the limited pore size and can only be released upon pH-responsive framework decomposition. For example, using ZIF-8 as a carrier and silk fibroin as a biological template, the carrier size was tuned to 100–150 nm. DOX and indocyanine green (ICG) were efficiently encapsulated via a one-pot method, with drug encapsulation efficiency exceeding 95% (Figure 5b).^105^ The platform was further modified with the MCF-7 breast tumor-targeting peptide (AR peptide, AREYGTRFSLIGGYR). Upon intravenous injection, the MOF particles could specifically target breast tumors to achieve excellent antitumor effects through ICG-assisted PDT, PTT, and DOX-based chemotherapy. In another example, Kartogenin (KGN), which promotes cartilage regeneration, was encapsulated into the ZIF-8 carriers using a one-pot method (Figure 5b). The carrier was further modified with HA to construct the KZIF@HA platform.^106^ HA enabled targeted adhesion and structural self-reinforcement of cartilage, achieving acid-responsive and stable drug release for up to 4 weeks, and thus significantly improving cartilage penetration and joint retention.

ZIF-67 and ZIF-8 are common in SOD topology and organic ligands. Both can be synthesized at room temperature, and are capable of acid-responsive degradation for drug release.^107^ Li and coworkers loaded palbociclib (Pal) and gallium ions (Ga^3+^, a ferroptosis inducer) onto the ZIF-67 platform *via* an *in situ* encapsulation strategy (Figure 5c).^108^ The Pal/ZIF-67 nanoparticles exhibited a uniform flower-like structure with a particle size of ∼300 nm. Using X-ray photoelectron spectroscopy and X-ray diffraction confirmed the incorporation of Ga^3+^ and the loading of Pal into ZIF-67. While ZIF-67 remained stable at physiological pH, 75% of Pal was released from the MOF within 24 h in an acidic environment (pH 5.0). This platform selectively induced senescence in tumor cells with high CDK4/6 activity, thereby sensitizing them to undergo ferroptosis, enhancing antitumor efficacy while protecting normal cells.

ZIF-90, as another MOF material with mild synthetic conditions, can be used to construct ATP-response drug delivery systems. For example, AB680 (a CD73 inhibitor) was *in situ* encapsulated during the preparation of ZIF-90 using a one-pot method (Figure 5d).^109^ ZIF-90 exhibited minimal drug leakage under physiological conditions. Upon treatment with 1 mM ATP, the MOF rapidly disintegrated to set free AB680. This increase was attributed to the stronger coordination between ATP and Zn^2+^ than that between ICA and Zn^2+^, which resulted in degradation of ZIF-90. In 4T1 tumor-bearing mice, treatment with AB680/ZIF-90 achieved a 37.2% inhibitory rate against tumoral CD73 expression and markedly curtailed adenosine production. When further combined with anti-PD-1 immunotherapy, the survival time of tumor-bearing mice was prolonged to 100 days.

Furthermore, the imidazole groups on the side chains of histidine can coordinate with Zn^2+^ to construct MOF delivery systems. For example, a pH-responsive nanoplatform EG/PZA was constructed by using a hexahistidine peptide (His6) as organic ligand and Zn^2+^ as metal node in the presence of ethyl gallate (EG) in an aqueous solution at room temperature, followed by 15 min of sonication (Figure 5e).^110^ At pH 6.0, EG/PZA could maintain the EG concentration within the active range for up to 7 days. *In vivo* experiments indicated that EG/PZA effectively promoted functional recovery in osteoarthritic mice, confirming its potential for the treatment of osteoarthritis and other chronic tissue injuries induced by ROS.

Despite these advances, the *in situ* encapsulation strategy still has the following limitations: (1) Only limited small-molecule drugs (*e.g.,* camptothecin,^111^ DOX,^112^ caffeine,^113^) bearing specific functional groups (*e.g.,* −COOH, −SO_3_H) or those with opposite charges can be efficiently encapsulated into MOF. For instance, the chemotherapeutic drug cytarabine (Ara) lacks the above-mentioned properties and thus cannot be effectively encapsulated into MOF. To improve drug-loading efficiency, an Ara prodrug (Ara-IR820) was prepared by covalently coupling Ara to indocyanine green (IR820). The −SO_3_H group in Ara-IR820 facilitated interaction of the conjugate with ZIF-8, with a drug loading of up to 39.8 wt % (Figure 5f).^114^ (2) Although a sufficient level of drug loading is required for therapeutic efficacy, excessively high drug content may interfere with normal crystal nucleation and the growth of MOFs, leading to diminished particle crystallinity, impaired colloidal stability, and thus premature degradation or burst release of the system under physiological conditions.^115^ Future research should explore the synthesis of MOF systems under mild conditions (room temperature and atmospheric pressure), integrating ingenious drug-release mechanisms, and extending MOFs based on endogenous components. Notably, mechanochemical synthesis enables one-step construction of MOFs and *in situ* encapsulation of guest molecules within minutes at room temperature, offering a sustainable and scalable approach.^116^ For example, a recently developed one-pot mechanochemical method has proved successful for simultaneously introducing structurally diverse guest molecules including imidazole, urea, and sulfamic acid into a MOF, NENU-3,^117^ providing an ideal methodology for the *in situ* encapsulation of labile drugs.

### 2.5 Drug-Loaded MOFs Constructed by Coordination Chemistry

Small molecule drugs can participate in the construction of MOFs with different mechanisms (Figure 6a) including (1) directly participating in the construction of MOFs as organic ligands, (2) binding to the MOF framework as modulators or end-capping agents, (3) participating in the construction of MOFs as metal nodes (*e.g.,* metallodrugs), and (4) forming carrier-free MOFs (in this case metallodrugs are directly coordinated with another organic therapeutic used as the ligand). These MOFs are advantageous in terms of uniformity in drug distribution within MOFs with high drug-loading efficiency. The therapeutic properties of these specific MOFs are characterized by the gradual release of the active drug or prodrug ligands from the framework under a physiological environment. However, appropriate preparation methods need to be developed to avoid impairing the therapeutic activity of the drug/prodrug used as structural units.

**Figure 6:**
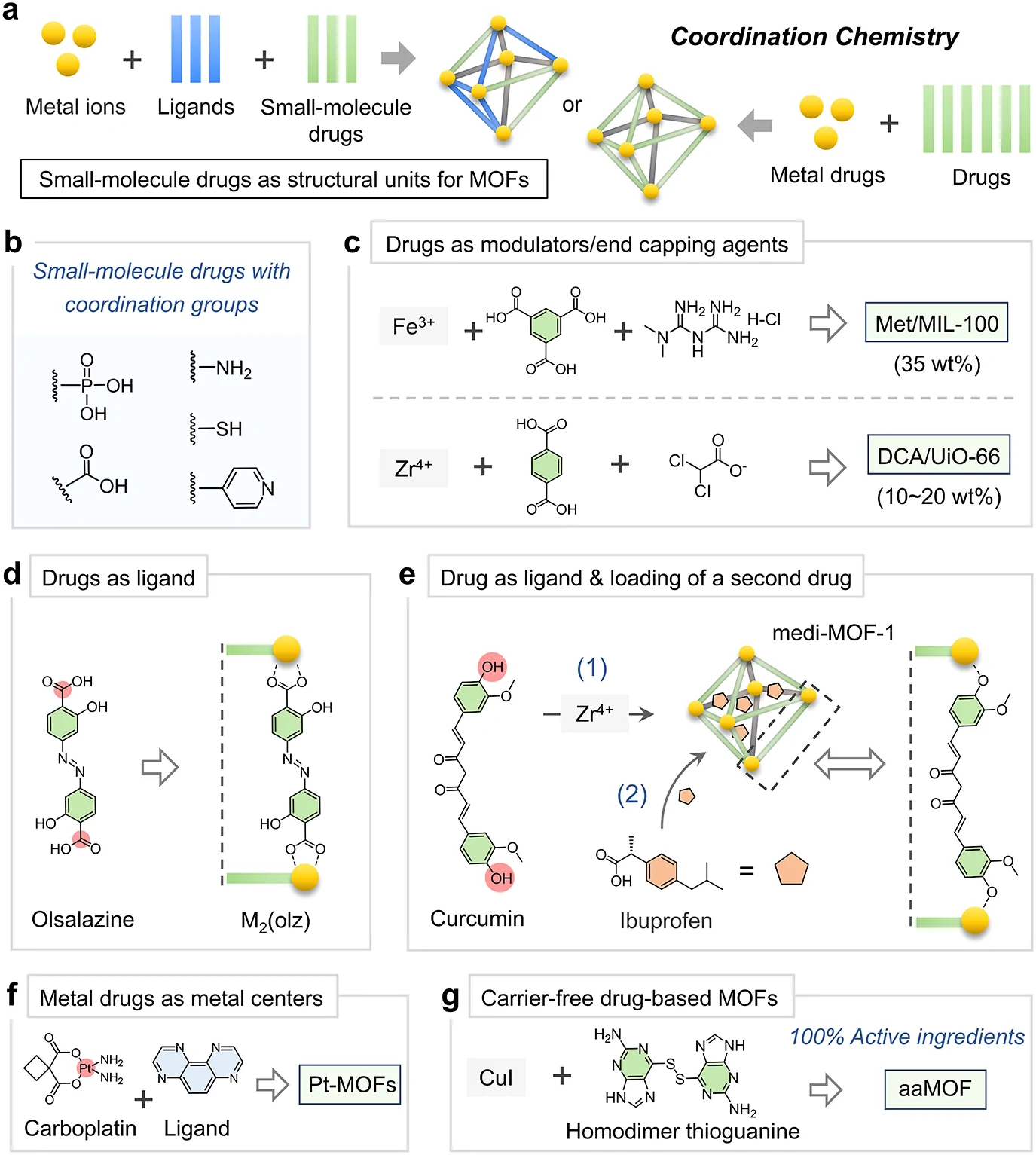
Schematic illustration of drug-loaded MOFs constructed by coordination chemistry. a) Design principle for loading drugs into MOFs *via* coordination chemistry. b) Representative coordination groups found in small-molecule drugs capable of participating in MOF formation.^118^ c) Construction of Met/MIL-100 hybrid material to improve metformin bioavailability and achieve pH-responsive drug release; construction of DCA/UiO-66 nanoparticles for selective antitumor cytotoxicity and immune system response.^120,121^ d) Olsalazine-based MOFs as a biocompatible platform for drug delivery.^123^ e) medi-MOF-1 nanoparticles, prepared by employing curcumin as the organic ligand, for pancreatic cancer therapy.^125^ f) Construction of padlock-designed Pt-MOFs and its application in promoting lymphocyte apoptosis and inhibiting metastasis in the central nervous system.^126^ g) An aaMOF formed by the coordination of CuI with thioguanine for GSH and pH dual-responsive cancer therapy.^127^

Drugs containing functional groups such as carboxyl, phosphonic, amino, thiol, and pyridyl groups can participate in the formation of MOFs through coordination interactions (Figure 6b).^118^ However, most drugs or prodrugs lack the coordination geometry or molecular rigidity required to construct crystalline MOFs, so they more commonly act as modulators or end-capping molecules. For example, metformin (Met,^119^ an oral hypoglycemic drug used in the treatment of type II diabetes) was used as a modulator in the construction of MIL-100 (Fe), with a drug-loading efficiency of 35 wt % (Figure 6c). After coating with sodium alginate, the system exhibited a gastrointestinal pH-responsive release behavior. While almost no release of drugs was detected in simulated gastric juice, a ∼87% cumulative release was found in a simulated intestinal environment over 8 h, thus implying a potentially improved oral bioavailability of Met.^120^ However, the efficacy of this platform has not been validated in animal models. In another example, Forgan and coworkers developed a coordination strategy that uses dichloroacetate (DCA), which has metabolic antitumor activity, as a regulator during the UiO-66 synthesis process. DCA can attach to the UiO-66 defect sites, with a loading capacity of 10–20 wt % (Figure 6c).^121^ After surface modification, its colloidal stability and resistance to phosphorylation degradation were enhanced, thus enabling highly efficient cytoplasmic delivery of DCA to kill cervical and breast cancer cells.

By selecting drugs with structural symmetry as ligands, they can be incorporated into MOFs during the self-assembly process, becoming an integral part of the MOF structure. At present, only a few MOFs can be constructed using drug molecules solely as bridging ligands, including olsalazine, azelaic acid, curcumin, and ellagic acid, where the drug mass accounts for 60%–80% of the total framework mass. For example, the anti-inflammatory drug oseltamivir (H_4_olz)^122^ was selected as the organic ligand to synthesize a novel extended MOF-74 family, denoted as M_2_(olz), where M = Fe, Co, Mg, Ni, and Zn (Figure 6d).^123^ The M_2_(olz) materials were the first series of isostructural bioactive frameworks exhibiting mesoporosity with a pore diameter of 27 Å. Mg_2_(olz) was shown to disassemble in physiological environment to release olsalazine, with an unprecedented drug loading capacity of 86 wt % for bioactive MOFs. In another example, gallic acid (GA), an antioxidant, was selected as the organic ligand and Mg^2+^ as the metal center to rapidly prepare Mg-GA MOF within 30 min via microwave-assisted synthesis.^124^ In physiological environments, phosphate competitively coordinated with Mg^2+^ to disrupt the Mg-GA coordination, thereby leading to a gradual release of GA. In a diabetic wound model, Mg-GA effectively regulated the oxidative stress microenvironment and macrophage polarization, promoting wound healing.

Large-pore drug-based MOFs constructed from drug molecules enable the loading of a second drug, thus facilitating the simultaneous delivery of multiple therapeutic agents. For example, a highly porous medi-MOF-1 composed of zinc and curcumin was used to deliver ibuprofen. medi-MOF-1 was not only used as a carrier, but also as an active pharmaceutical ingredient itself (Figure 6e).^125^ This material exhibited a permanent porous structure, a high BET surface area of 3002 m^2^/g, and a pore diameter of 11 Å. Drug loading of medi-MOF-1 was achieved by pore encapsulation, and the loaded drug could be released in a phosphate ion-responsive manner. medi-MOF-1 was unstable in PBS (pH 7.4), with 52.5% of ibuprofen being released within 2 h. Additionally, the biodegradability of medi-MOF-1 was investigated, and the degradation process of medi-MOF-1 proceeded rapidly within 1 h, releasing 30.5% of the total linker in the material. Finally, the results of the MTT evaluation indicated that curcumin released from the degradation of 50 μg mL^–1^ medi-MOF-1 effectively inhibited the proliferation of pancreatic cancer cells (BxPC-3).

In addition to the approach where drug molecules serve as organic ligands, metallic therapeutics can also be directly used as metal centers to construct therapeutic MOFs. For example, Miao and coworkers developed a carboplatin-based MOF (Pt-MOFs@Glu) for targeted treatment of central nervous system lymphoma (CNSL) (Figure 6f).^126^ Pt-MOFs@Glu consists of carboplatin as the metal center, pyrazinoquinoxaline as the molecular lock for carboplatin, and β-glucan as the central nervous system targeting and immune activation component. The carrier was stable in the gastrointestinal tract and circulation, and the high ROS in the tumor microenvironment triggered structural disintegration, achieving the avalanche release of carboplatin. In the orthotopically established mouse model of CNSL, oral administration of Pt-MOFs@Glu prolonged the survival of mice and reduced the tumor volume.

Carrier-free systems represent a promising research direction due to their extremely high drug loading capacity (up to 100%), mitigation of carrier-induced toxicity, and simple manufacturing process. Based on this concept, Lei and coworkers designed and synthesized an aaMOF using 6-thioguanine (6-TG) (a clinically used base-analog drug) as the ligand, and copper (I) iodide (CuI) as the metal node, achieving 100% active ingredient loading (Figure 6g).^127^ aaMOF was stable in normal physiological environments, but rapidly depolymerized in the acidic and high-GSH microenvironment of tumors, simultaneously releasing Cu^+^, I^–^, and 6-TG. aaMOF-based radiotherapy achieved a tumor inhibition rate of 66% in xenograft tumor models. Similarly, Li and coworkers constructed a hypoxia-specific MOF nanosystem (AFNMOFs) *via* the self-assembly of Fe^3+^, hypoxia-activated prodrug AQ4N, and the IDO-1 pathway inhibitor NLG919.^128^ AFNMOFs exhibited a uniform morphology with a particle size of ∼132 nm and distinct crystalline features. This carrier achieved high drug-loading efficiencies of up to 92.24% for AQ4N and 3.89% for NLG919. After treatment with high-intensity focused ultrasound (HIFU), the AFNMOFs significantly inhibited the growth of primary and distant tumors, reduced lung metastases, and reshaped the tumor immune microenvironment. The MOF also exhibited good *in vivo* biocompatibility.

Unlike other loading strategies, the strategy detailed in this section directly incorporates small-molecule drugs as integral components of the MOF at the molecular level, theoretically achieving the highest possible loading efficiency. Moreover, by adjusting the types of metal ions and drug ligands, the pore size of the MOF can be precisely controlled, enabling the loading of a second type of drug to construct a synergistic therapeutic system. However, a prerequisite for application is that the drug molecule must possess an effective coordination group, and the pharmacological activity must not be lost after coordination, which imposes high requirements on the structure of drug molecules.

## 3 Comparison of Strategies for Loading Small-Molecule Drugs into MOFs

As mentioned earlier, drug loading strategies for MOFs can be primarily categorized into the following five types: (1) surface adsorption, (2) covalent chemistry, (3) pore encapsulation, (4) *in situ* encapsulation, and (5) coordination chemistry. To guide the rational selection of drug-loading strategies, Table 1 summarizes the representative drug loading capacities, encapsulation efficiencies, release characteristics, and the main advantages and limitations of each strategy.

**Table 1:** Key Differences between Five Types of Drug Loading Strategies

Strategy	Surface Adsorption	Covalent Chemistry	Pore Encapsulation	*In-situ* Encapsulation	Coordination Chemistry
Drug Loading Capacity	High	Moderate	Moderate to high	High	Highest (up to 100 wt %)
Preparation Complexity	Simple (one-step mixing)	Complex (multistep synthesis)	Moderate (impregnation/grinding)	Moderate (one-pot synthesis)	Moderate to Complex
Drug Location	MOF surface	MOF surface	Within pore channels	MOF interior	MOF interior or surface
Drug-MOF Interaction	Weak (noncovalent)	Strong (covalent bonds)	Moderate (host–guest)	Moderate–Strong	Strong (coordination bonds)
Release Profile	Fast; prone to burst release	Stimuli-cleavable linkers enable on-demand release	Sustained release over hours to days	pH/ATP-responsive	Framework degradation-triggered
Key Advantages	Simplicity; preserves drug activity; rapid formulation	Precise release control; minimal leakage; prodrug compatibility	Physical protection of cargo; tailorable pore microenvironment;	Uniform distribution; high efficiency; overcomes pore limitations	Maximum loading; carrier-free potential
Primary Limitations	Weak binding; premature release *in vivo*	Limited drug scope; complex procedures	Pore size constraints	Limited to mild-synthesis MOFs	Strict drug structural requirements

From the perspective of the inherent characteristics of small-molecule drugs, charge, functional group types, molecular size, and stability of these drugs are key considerations for strategy selection. (1) For drugs containing coordinating groups (*e.g.,* −COOH, pyridyl, phenolic hydroxyl groups) and structural symmetry, coordination-chemistry strategies are preferred. Such drugs can be directly used as ligands to construct MOFs, achieving an ultrahigh drug loading capacity and even carrier-free fully active systems. (2) For drugs with relatively small molecular size and abundant modifiable side chains (*e.g.,* −NH_2_ and −COOH groups), covalent chemistry can be used to introduce stimulus-responsive linkers for controlled release. If the molecular size is smaller than the pore size of the MOF, pore encapsulation can also be used to achieve sustained diffusion release. (3) For neutral/hydrophobic drugs with molecular dimensions that are matched to MOF pore size, pore encapsulation can be used, leveraging hydrophobic cavities or functionalized channels to achieve high loading and sustained release. (4) Ionic drugs with large molecular sizes and stable chemical properties are suitable candidates for the surface adsorption strategy, which achieves efficient drug loading via electrostatic interactions. (5) For drugs with large molecular size, easy degradation and inactivation, that exhibit difficulty to achieve efficient loading through pore encapsulation, the *in situ* encapsulation strategy can be used to integrate the drug into the MOFs during framework formation to achieve uniform drug loading.

For different biomedical applications, different loading strategies should be selected. (1) For superficial lesions such as wound infection, the surface adsorption strategy is preferred. MOFs can also be combined with hydrogels, medical dressings, or functional coatings to achieve sustained drug release.^129^ (2) For systemic drug delivery requiring long circulation and targeted controlled release, the covalent chemical strategy should be chosen because of its high circulation stability and capacity for stimuli-responsive drug release in the tumor microenvironment. (3) For *in vivo* drug delivery that requires physical barrier protection for drugs and stable long-term sustained release, the pore encapsulation or *in situ* encapsulation strategy should be chosen to effectively avoid premature drug degradation sustaining a long release period. (4) In pursuit of the highest possible drug loading capacity and optimization of carrier toxicity, the carrier-free MOF system based on coordination chemistry (100% active drug components) is the optimal choice, which has unique advantages in the development of carrier-free nanomedicines.

In practical applications, the above strategies should be flexibly selected or combined according to the molecular structure of drugs, delivery scenario, and therapeutic requirements, to advance the clinical translation of MOF-based drug delivery systems through rationally selecting a design strategy.

## 4 Conclusions

Currently, research achievements of MOF carriers have remained at the theoretical stage, with no genuine applications seen in clinical trials or marketed formulations of pharmaceutical preparations, and there is still a considerable gap before practical translation.^130^ This perspective focuses on analyzing the challenges faced by MOFs as delivery carriers for small-molecule drugs and their future development directions, to promote their translation into clinical applications.

1 **Biocompatibility.** The selection of appropriate metal ions and organic linkers is the primary consideration for ensuring the biocompatibility and safety of MOF carriers, including the use of endogenous metal ions (*e.g.,* Fe^3+^, Zn^2+^, Ca^2+^, Mg^2+^) and biocompatible organic ligands (*e.g.,* amino acids, cyclodextrins). Furthermore, MOFs should be rationally engineered to ensure degradation with well-controlled kinetics and pathways, enabling their complete decomposition into nontoxic products that can be readily excreted , thereby avoiding long-term accumulation and thus the associated adverse effects.^131,132^

2 **Rational design and performance prediction driven by AI.** For MOF systems with high chemical diversity, we encourage researches to use advanced technical approaches for rational design and precise screening, to discover materials with enhanced potential for specific applications. For example, AI can be used to predict MOFs with optimal drug compatibility and loading efficiency, and also to predict drug release behavior and *in vivo* distribution characteristics, thereby shortening the research and development cycle and reducing the cost associated with simple trial and error approaches.
3 **In vivo stability and targeting ability.** Regardless of the loading strategy adopted, MOFs tend to dissociate or undergo nonspecific degradation in physiological environments, resulting in premature drug release before reaching the target site. To address this issue, the colloidal stability of drug-loaded MOF formulations can be improved through surface functionalization and the regulation of particle size and surface charge. For example, coating MOF with polymers (such as polyethylene glycol, polydopamine, polyvinylpyrrolidone, chitosan, and sodium alginate), biomolecules, or even intact cell membranes can not only improve the structural stability of MOFs under physiological conditions, but also prevent premature drug release and enhance biocompatibility.^133^
4 **Standardization, scalable manufacturing, and batch-to-batch reproducibility.** The clinical translation of MOFs relies on stable and scalable preparation processes. Currently, small-scale laboratory synthesis can not meet the batch requirements for clinical research. Scale-up production often leads to problems such as impure crystal forms, uneven particle size distribution, and significant batch-to-batch differences in physicochemical properties. Moreover, scalability should be considered together with sustainability, as the development of eco-friendly and energy-efficient synthetic routes is essential for reducing environmental burden and ensuring the long-term viability of MOF-based biomedical applications. As such, it is necessary to establish standardized synthetic and characterization approaches, together with green manufacturing methods, to promote the transition from small-scale laboratory synthesis to large-scale production in compliance with good manufacturing practice, ensuring high batch-to-batch consistency and quality controllability of the materials.

In the future, the interdisciplinary integration of materials science, pharmacy, medicine, AI, and other fields is expected to further advance the transition of MOF carriers from laboratory research to clinical usage. Meanwhile, we anticipate that MOFs will not only serve as drug carriers but also exert therapeutic functions to achieve synergistic effects with drugs. We believe that the development of MOFs with active components represents a pivotal direction requiring sustained attention to deliver the full potential of MOF-based therapeutics.
